# Mucilaginibacter aureus sp. nov. and Mucilaginibacter sediminis sp. nov., isolated from wetland soil

**DOI:** 10.1099/ijsem.0.007042

**Published:** 2026-01-22

**Authors:** Chae Yeong Moon, Jae Kyeong Lee, Dong Min Han, Dae Seung Lee, Byeong Jun Choi, Ju Hye Baek, Che Ok Jeon

**Affiliations:** 1 Department of Life Science, Chung-Ang University, Seoul 06974, Republic of Korea

**Keywords:** *Bacteroidota*, *Mucilaginibacter aureus*, *Mucilaginibacter sediminis*, new taxa, wetland

## Abstract

Two Gram-stain-negative, non-motile, rod-shaped bacterial strains, designated AW1-3^T^ (strictly aerobic) and AW1-7 ^T^ (facultatively aerobic), both catalase- and oxidase-positive, were isolated from a reed wetland in South Korea. Strain AW1-3^T^ grew at 15–35 °C, pH 6.0–7.0 and in 0.0–0.5% (w/v) NaCl, whereas strain AW1-7^T^ grew at 10–35 °C, pH 6.0–7.0 and in 0–0.5% NaCl. The major fatty acids (>10%) were iso-C_15:0_ and summed feature 3 (C_16:1_* ω*7*c* and/or C_16 :1_* *ω*6c*) in strain AW1-3^T^ and C_16:0_, summed feature 3, C_18:0_ and iso-C_15:0_ in strain AW1-7^T^. Phosphatidylethanolamine was the major polar lipid in both strains, and menaquinone-7 was the sole respiratory quinone. The genomic DNA G+C contents were 43.9 mol% (AW1-3^T^) and 43.2 mol% (AW1-7^T^). The two strains shared 94.9% 16S rRNA gene sequence similarity, with ANI and dDDH values of 70.9% and 20.0%, respectively, indicating they represent distinct species. Phylogenetic and phylogenomic analyses based on 16S rRNA gene and whole-genome sequences placed strains AW1-3^T^ and AW1-7^T^ in close association with *Mucilaginibacter rivuli* HMF5004ᵀ and *Mucilaginibacter ginsenosidivorax* KHI28ᵀ, respectively. However, strain AW1-3^T^ shared 16S rRNA gene sequence similarity/ANI/dDDH values of 97.7%/73.7%/19.8% with *M. rivuli* HMF5004^T^, and strain AW1-7ᵀ shared 98.5%/88.6%/36.6% with *M. ginsenosidivorax* KHI28ᵀ, all below the species delineation thresholds, supporting their recognition as novel species. Based on phenotypic, chemotaxonomic and genomic characteristics, the names *Mucilaginibacter aureus* sp. nov. (AW1-3^T^=KACC 23848^T^=JCM 37500^T^) and *Mucilaginibacter sediminis* sp. nov. (AW1-7^T^=KACC 23849^T^=JCM 37501^T^) are proposed.

## Introduction

The genus *Mucilaginibacter*, classified within the family *Sphingobacteriaceae* of the phylum *Bacteroidota*, was first proposed in 2007 to accommodate *Mucilaginibacter paludis*, the type species, which was isolated from an acidic sphagnum peat bog and shown to degrade various polysaccharides, including pectin, xylan and laminarin [1]. Since then, the genus has been emended through several revised descriptions [2,4]. As of 7 August 2025, the genus comprises 81 validly published and 11 invalidly published species (https://lpsn.dsmz.de/genus/mucilaginibacter). Members of *Mucilaginibacter* have been isolated from diverse environments, including soil [5,7], rotten wood [8,9], flowers [10], glacier ice [11], freshwater sources [12,14], rice straw [15], wood chips [16], the rhizosphere of *Platycodon grandiflorum* [17] and wetland water [18]. Members of this genus are generally characterized as Gram-stain-negative, non-motile, rod-shaped organisms that typically exhibit catalase and oxidase activities [1,18]. They possess menaquinone-7 (MK-7) as the major respiratory quinone, iso-C_15:0_ and summed feature 3 (C_16:1_* *ω*7c* and/or C_16:1_* *ω*6c*) as major cellular fatty acids and phosphatidylethanolamine (PE) as the major polar lipid. In the present study, two putative novel strains affiliated with the genus *Mucilaginibacter* were isolated from soil sediment collected from a reed wetland, and their taxonomic characteristics were investigated using a polyphasic approach.

## Strain isolation

Strains AW1-3^T^ and AW1-7^T^ were isolated from soil sediment collected from a reed wetland in Ansan City, Republic of Korea (37° 16′ 29.7″ N, 126° 50′ 24.0″ E), as previously described [16]. Briefly, sediment samples were resuspended and serially diluted in PBS (137 mM NaCl, 2.7 mM KCl, 10 mM Na_2_ HPO_4_ and 2 mM KH_2_PO_4_, pH 7.2). Aliquots (100 µl) of each dilution were spread onto Reasoner’s 2A (R2A) agar (MBcell, South Korea) and incubated aerobically at 25 °C for 2 days. Colonies grown on R2A agar were resuspended in 100 µl of 5% (w/v) Chelex-100 solution (Bio-Rad) and boiled for 10 min to prepare crude genomic DNA lysates. The 16S rRNA genes were amplified using universal primers 27F (5′-AGA GTT TGA TCM TGG CTC AG-3′) and 1492R (5′-TAC GGY TAC CTT GTT ACG ACT T-3′) [19]. PCR amplicons were digested with HaeIII and HhaI restriction enzymes and separated by electrophoresis on 2% agarose gels. Representative amplicons with distinct restriction fragment profiles were sequenced using the universal primer 340F (5′-CCT ACG GGA GGC AGC AG-3′) [19]. The resulting partial 16S rRNA gene sequences were compared with those of type strains using the EzBioCloud server (https://www.ezbiocloud.net/identify) [20]. Based on the sequence comparisons, two putatively novel strains, AW1-3^T^ and AW1-7^T^, affiliated with the genus *Mucilaginibacter*, were selected for further taxonomic characterization. The strains were routinely cultivated on R2A agar at 30 °C for 2 days and preserved in R2A broth supplemented with 15% (v/v) glycerol at –80 °C. The type strains *Mucilaginibacter rivuli* KCTC 82633ᵀ and *Mucilaginibacter ginsenosidivorax* KACC 14955ᵀ, which showed the highest 16S rRNA gene sequence similarity to strains AW1-3^T^ and AW1-7^T^, respectively, were obtained from culture collections and used as reference strains for phenotypic, chemotaxonomic and fatty acid composition comparisons.

## Phylogeny based on 16S rRNA gene sequences

The 16S rRNA gene amplicons of strains AW1-3^T^ and AW1-7^T^, initially amplified using primers 27F and 1492R, were further sequenced with universal primers 518R (5′-ATT ACC GCG GCT GCT GG-3′) and 805F (5′-GAT TAG ATA CCC TGG TAG TC-3′) [19]. Nearly complete 16S rRNA gene sequences were obtained for strains AW1-3^T^ (1,444 bp) and AW1-7^T^ (1,446 bp) by assembling reads from primers 340F, 518R and 805F. Sequence similarities between the two strains and their closely related type strains were calculated using EzBioCloud (http://www.ezbiocloud.net/identify) [20]. Multiple sequence alignments of the 16S rRNA gene sequences of strains AW1-3^T^ and AW1-7^T^ and related type strains were performed using Infernal (version 1.1.4) [21]. Phylogenetic trees were reconstructed using the neighbour-joining (NJ), maximum parsimony (MP) and maximum likelihood (ML) methods in mega11 [22], with bootstrap values calculated from 1,000 replicates. The Kimura two-parameter model, nearest-neighbour-interchange heuristic search and subtree-pruning-and-regrafting algorithm were applied to construct the NJ, ML and MP trees, respectively.

**Fig. 1.**
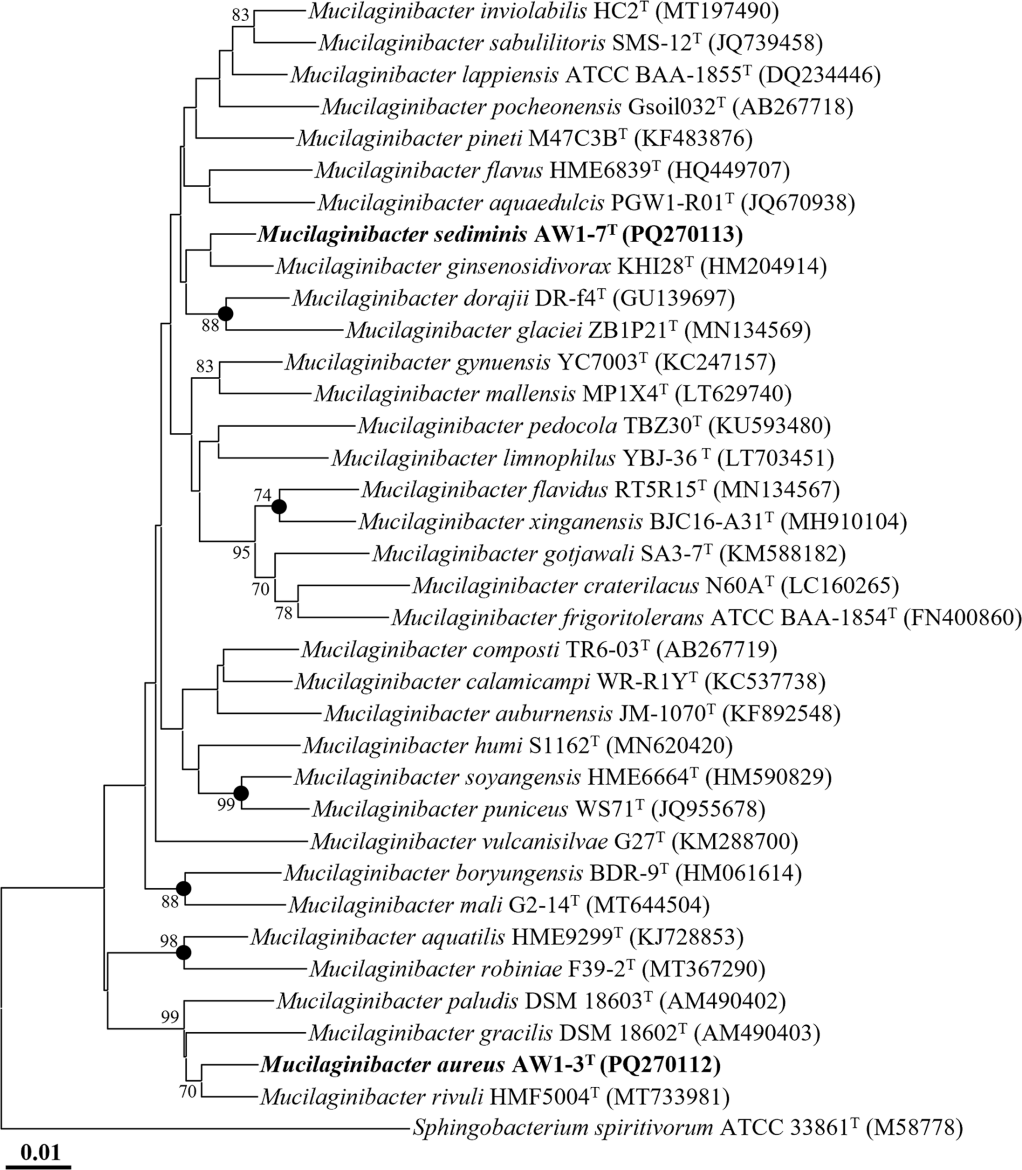
NJ tree showing the phylogenetic relationships of strains AW1-3^T^ and AW1-7^T^ with closely related taxa based on 16S rRNA gene sequences. Bootstrap values greater than 70% (based on 1,000 replicates) are indicated at the corresponding nodes. Filled circles (●) denote nodes also supported by ML and MP algorithms. *Sphingobacterium spiritivorum* ATCC 33861^T^ (M58778) was used as the outgroup. The scale bar indicates 0.01 nucleotide substitutions per site.

The 16S rRNA gene sequence similarity between strains AW1-3^T^ and AW1-7^T^ was 94.9%, which is well below the commonly accepted species delineation threshold of ~98.6% [23], indicating that the two strains likely represent distinct species. Further comparative analysis showed that strain AW1-3^T^ shared the highest sequence similarity with *M. rivuli* HMF5004ᵀ (97.7%), while strain AW1-7^T^ was most closely related to *M. ginsenosidivorax* KHI28ᵀ (98.5%), supporting their classification as novel species within the genus *Mucilaginibacter*. Phylogenetic analysis based on 16S rRNA gene sequences using the NJ algorithm showed that strains AW1-3^T^ and AW1-7^T^ clustered with *M. rivuli* HMF5004ᵀ and *M. ginsenosidivorax* KHI28ᵀ, respectively, within the genus *Mucilaginibacter* (Fig. 1). This clustering was also supported by ML and MP analyses (Fig. S1, available in the online Supplementary Material). These combined phylogenetic and comparative results strongly suggest that strains AW1-3^T^ and AW1-7^T^ represent novel species within the genus *Mucilaginibacter*.

## Ecological distribution analysis

To examine the ecological distributions of strains AW1-3^T^ and AW1-7^T^, their 16S rRNA gene sequences were compared against 500,048 metagenomic 16S rRNA amplicon datasets derived from diverse environments, including soil, plants, crops, seawater, freshwater, marine and freshwater sediments, algae, crustaceans, sand, biofilms, food, skin and gut, using the Integrated Microbial Next-Generation Sequencing (IMNGS) platform [24] at a 99.0% sequence similarity threshold.

The IMNGS analysis revealed that both strains were broadly distributed in various plant- and insect-associated environments (Table S1). Strain AW1-3^T^ was majorly detected across various plant-related metagenomes, including those of *Ginkgo*, *Zea mays*, *Hordeum vulgare*, *Glechoma hederacea*, *Boechera stricta* and leaf litter, showing the highest prevalence in the *Ginkgo* metagenome (62.9%) with a very high average relative abundance (ARA) of 2.9%. Strain AW1-7^T^ was also detected in several plant-associated metagenomes but displayed a little narrower distribution and lower abundance, indicating subtle niche differentiation between the two strains. Although both strains were originally isolated from wetland soil, they appeared in soil metagenomes at relatively low prevalence and abundance. In addition, sequences corresponding to both strains were found in several insect- and animal-associated metagenomes, including *Cancer borealis*, *Onthophagus similis*, *Crioceris duodecimpunctata*, *Rhipicephalus sanguineus* and *Bos taurus*, albeit at low prevalence. These findings suggest that plant-associated environments may represent the primary ecological habitats of strains AW1-3^T^ and AW1-7^T^, while their presence in soil and animal-related metagenomes likely reflects indirect associations through dietary or environmental exposure.

## Whole-genome sequencing and genome-based phylogeny

Genomic DNA from strains AW1-3^T^ and AW1-7^T^ was extracted from cells cultured in R2A broth using the Wizard Genomic DNA Purification Kit (Promega, USA), following the manufacturer’s instructions. Whole-genome sequencing was performed in-house using the Oxford Nanopore MinION platform (ONT, UK). The resulting reads were *de novo* assembled with Flye v2.9.1 (nanohq option) [25] and subsequently polished with subsampled reads using the built-in Flye polisher. Genome quality was evaluated with CheckM2 (version 1.0.2) [26], based on completeness and contamination metrics. Phylogenomic analysis of strains AW1-3^T^ and AW1-7^T^, along with closely related type strains, was conducted using the Genome Taxonomy Database Toolkit (GTDB-Tk) based on 120 ubiquitous single-copy marker genes (bac120 marker set) [27]. An ML phylogenomic tree was constructed from the concatenated amino acid sequences of these marker genes using mega11, with bootstrap support from 1,000 replicates. Genome relatedness was further assessed by calculating average nucleotide identity (ANI) and digital DNA–DNA hybridization (dDDH) values using the Orthologous ANI Tool available on the EzBioCloud server (https://www.ezbiocloud.net/tools/orthoani) [28] and the Genome-to-Genome Distance Calculator (version 2.1; https://ggdc.dsmz.de/distcalc2.php) [29], respectively.

**Fig. 2.**
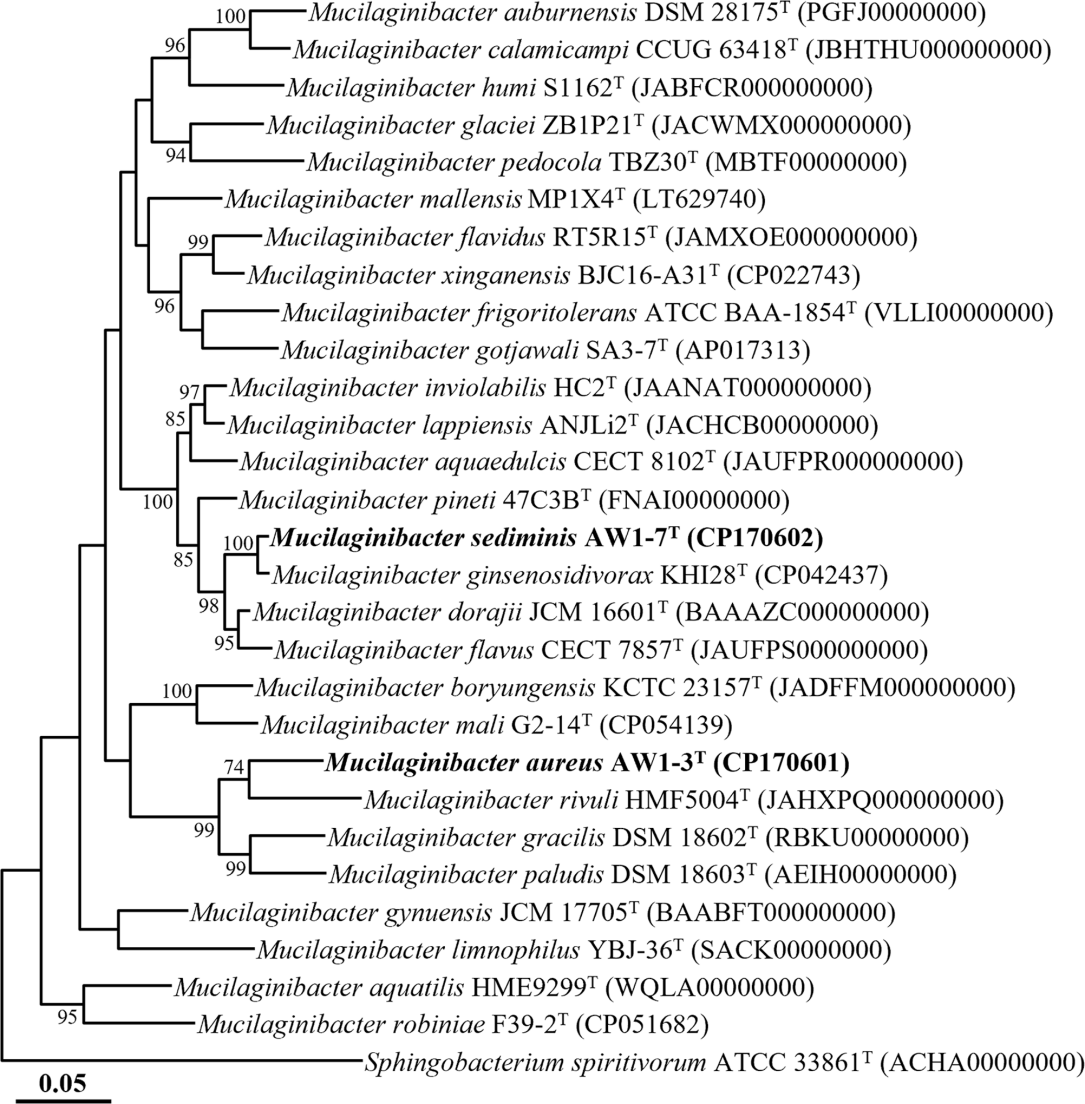
Phylogenomic tree showing the relationships of strains AW1-3^T^ and AW1-7^T^ with closely related taxa, based on concatenated protein sequences of the 120 bacterial marker genes (bac120) from GTDB-Tk. Bootstrap values above 70% (from 1,000 replicates) are indicated at the corresponding nodes. *Sphingobacterium spiritivorum* ATCC 33861^T^ (ACHA00000000) was used as the outgroup. The scale bar represents 0.05 substitutions per amino acid position.

*De novo* assembly of MinION sequencing data produced complete genomes for strains AW1-3^T^ and AW1-7^T^, measuring 5,509 kb and 7,410 kb, respectively, each consisting of a single circular chromosome. CheckM2 analysis confirmed their high quality, with completeness and contamination values of 100% and 0.23% for strain AW1-3^T^ and 99.99% and 0.35% for strain AW1-7^T^, respectively, satisfying the standard criteria for high-quality genomes (completeness ≥90% and contamination ≤10%) [26]. The 16S rRNA gene sequences identified in the genomes were identical to those obtained through PCR-based sequencing. Phylogenomic analysis revealed that strains AW1-3^T^ and AW1-7^T^ formed distinct and well-supported lineages with *M. rivuli* HMF5004^T^ and *M. ginsenosidivorax* KHI28^T^, respectively, within the genus *Mucilaginibacter* (Fig. 2), consistent with the 16S rRNA gene-based phylogeny. The ANI and dDDH values between AW1-3^T^ and AW1-7^T^ were 70.9% and 20.0%, respectively, well below the species delineation thresholds (ANI~95%, dDDH 70%) [23], indicating that the two strains represent distinct species. Furthermore, strain AW1-3^T^ shared ANI and dDDH values of 73.7% and 19.8% with *M. rivuli* HMF5004^T^, while strain AW1-7^T^ showed 88.6% and 36.6% values with *M. ginsenosidivorax* KHI28^T^, respectively (Table S2). These values also fall significantly below the accepted thresholds for species demarcation. Collectively, the phylogenomic and genome similarity analyses strongly support that strains AW1-3^T^ and AW1-7^T^ represent two novel species within the genus *Mucilaginibacter*.

## Genomic features and CAZyme gene analysis

The whole-genome sequences of strains AW1-3^T^ and AW1-7^T^ were deposited in the GenBank database and annotated using the NCBI (National Center for Biotechnology Information)

Prokaryotic Genome Annotation Pipeline. Carbohydrate-active enzymes (CAZys) were identified using the dbCAN3 meta server (https://bcb.unl.edu/dbCAN2/) [30]. The genome of strain AW1-3^T^ comprises 4,760 total genes, including 4,694 protein-coding sequences, 2 rRNA operons (16S, 23S and 5S) and 49 tRNA genes. In contrast, the genome of strain AW1-7^T^ contains 6,173 total genes, including 6,088 protein-coding sequences, 3 rRNA operons and 53 tRNA genes (Table 1). Although the two strains differ markedly in genomic features such as genome size and gene content, these values fall within the range observed in other *Mucilaginibacter* species (Table 1), highlighting high genomic diversity within the genus. The DNA G+C contents of strains AW1-3^T^ and AW1-7^T^ were 43.9 mol% and 43.2 mol%, respectively, consistent with those reported for other *Mucilaginibacter* species [1,18].

**Table 1.** General genomic features of strain AW1-3^T^ and AW1-7^T^ and closely related type strains of *Mucilaginibacter* Taxa: 1, strain AW1-3^T^ (CP170601); 2, *M. rivuli* HMF5004^T^ (JAHXPQ000000000); 3. strain AW1-7^T^ (CP170602); 4. *M. ginsenosidivorax* KHI28^T^ (CP042437).

Feature*	1	2	3	4
Genome status† (no. of contigs)	C (1)	D (14)	C (1)	C (1)
Total genome size (kb)	5,509	4,635	7,410	7,811
G+C content (mol%)	43.9	42.5	43.2	43.0
No. of total genes	4,760	3,950	6,173	6,390
No. of protein-coding sequences	4,694	3,897	6,088	6,249
No. of total RNA genes	58	46	65	67
No. of tRNA genes	49	40	53	55
No. of rRNA (16S, 23S, 5S) operons	2	1	3	3
No. of pseudogenes	8	7	20	74
No. of total CAZy† genes	305	262	391	411
Polysaccharide lyase	26	24	10	5
Carbohydrate esterase	29	24	29	25
Glycoside hydrolase	182	155	243	275
Glycosyltransferase	51	38	70	67
Carbohydrate-binding module	17	21	39	39

* The genomic features, excluding CAZy genes, were analysed using the NCBI prokaryotic genome annotation pipeline (https://www.ncbi.nlm.nih.gov/refseq/annotation_prok/).

† C, complete; D, draft.

Polysaccharides are among the most abundant organic nutrients in natural environments, and *Mucilaginibacter* species are recognized for their broad capacity to degrade diverse polysaccharides [1,8, 9, 15]. To evaluate the polysaccharide-degrading potential of strains AW1-3^T^ and AW1-7^T^, their genomes were analysed for CAZy genes. The analysis revealed that strains AW1-3^T^ and AW1-7^T^ encode 305 and 391 CAZy-related genes, respectively, numbers comparable to those of closely related *Mucilaginibacter* species (Table 1). The abundance of CAZy genes in these strains is notably higher than that in many other bacterial taxa. For instance, *Phycobium rhodophyticola*, isolated from algae, possesses only 52 CAZy genes [31], and members of the genus *Roseovarius* from red algae, including *Roseovarius phycicola* and *Roseovarius rhodophyticola*, also harbour only 63 and 59 CAZy genes, respectively [32]. Similarly, *Yoonia* strains such as *Yoonia algicola*, *Yoonia rhodophyticola* and *Yoonia phaeophyticola* contain only 71, 87 and 81 CAZy genes, respectively [33], and the soil bacterium *Microvirga terrae* carries 139 CAZy genes [34]. Collectively, these comparisons highlight that *Mucilaginibacter* species possess substantially higher CAZy gene counts, underscoring their remarkable versatility in polysaccharide degradation.

## Growth, morphology and physiology properties

The growth of strains AW1-3^T^ and AW1-7^T^ was evaluated on various agar media (all from MBcell), including marine agar (MA), R2A agar, Luria–Bertani (LB) agar, tryptic soy agar (TSA) and nutrient agar (NA), following incubation at 30 °C for 2 days. Growth was further assessed on R2A agar across a temperature range of 10–45 °C (in 5 °C intervals) and in R2A broth adjusted to pH values from 4.0 to 10.0 (in 1.0-unit increments), with incubation at 30 °C for 2 days. pH-adjusted media were prepared using sodium citrate (pH 4.0–5.0), sodium phosphate (pH 6.0–8.0) and sodium carbonate–bicarbonate (pH 9.0–10.0) buffers, with final pH corrections made after autoclaving if necessary. Salt tolerance was tested in R2A broth supplemented with NaCl concentrations ranging from 0 to 5% (w/v), in 0.5.% increments, incubated at 30 °C for 2 days.

Cellular morphology and motility of strains AW1-3ᵀ and AW1-7ᵀ were examined using phase-contrast microscopy (Zeiss Axio Scope.A1, Carl Zeiss, Germany). For detailed visualization, including flagellar structure, cells were placed on Formvar-coated copper grids, negatively stained with 2% (w/v) uranyl acetate (Sigma-Aldrich, USA) for 15 s and observed using a transmission electron microscope (JEM-1010, JEOL, Japan). Gliding motility was further evaluated by stab-inoculating R2A semi-solid agar (0.3%, w/v) and incubating at 30 °C. Gram staining was conducted using a commercial kit (bioMérieux, France) following the manufacturer’s instructions. Catalase activity was determined by bubble formation upon exposure to 3% (v/v) hydrogen peroxide (Junsei, Japan), and oxidase activity was assessed using 1% (w/v) tetramethyl-*p*-phenylenediamine (Merck, USA) [35]. Anaerobic growth was evaluated on R2A agar after 21 days of incubation at 30 °C under anaerobic conditions generated by the GasPak Plus system (BBL, USA). Phenotypic characteristics of strains AW1-3^T^ and AW1-7^T^ were compared with those of reference strains under identical conditions at their respective optimal growth temperatures. Hydrolysis of tyrosine, casein, aesculin, starch, Tween 20 and Tween 80 was tested on R2A agar following previously described protocols [35]. Additional biochemical characteristics were examined using API 20NE kits (bioMérieux, France) in accordance with the manufacturer’s instructions.

Strains AW1-3^T^ and AW1-7^T^ exhibited robust growth on R2A agar, slow growth on TSA and NA and no growth on MA and LB agar. Cells of both strains were Gram-stain-negative, non-motile rods lacking flagella, measuring 0.6–1.0 µm in width and 3.0–3.5 µm in length for strain AW1-3^T^ and 0.7–0.9 µm in width and 2.0–2.5 µm in length for strain AW1-7^T^ (Fig. S2). Strain AW1-3^T^ did not grow under anaerobic conditions, indicating an obligately aerobic nature, whereas strain AW1-7^T^ was capable of anaerobic growth, suggesting it is facultatively aerobic (Table 2). Both strains shared several phenotypic traits with reference strains of the genus *Mucilaginibacter*, including rod-shaped morphology; indole production; glucose fermentation; catalase, oxidase, *β*-galactosidase and urease activities; assimilation of caprate, citrate and phenylacetate; and hydrolysis of aesculin, Tween 20 and tyrosine. However, they differed from closely related *Mucilaginibacter* species in certain traits, such as growth range, gelatin hydrolysis and d-mannitol assimilation (Table 2).

**Table 2.** Comparison of differential phenotypic characteristics between strains AW1-3^T^ and AW1-7^T^ and their closely related *Mucilaginibacter* type strains Taxa: 1, strain AW1-3^T^ (this study); 2, *M. rivuli* KCTC 82633^T^ [13]; 3, strain AW1-7^T^ (this study); 4. *M. ginsenosidivorax* KACC 14955^T^ [14]. All strains are rod-shaped and positive for the following characteristics: activity* of catalase, oxidase, *β*-glucosidase and *β*-galactosidase and hydrolysis* of aesculin. All strains are negative for the following characteristics: Gram-staining, flagellum motility, indole production*, glucose fermentation*, urease activity*, hydrolysis* of Tween 20 and tyrosine and assimilation* of caprate, citrate and phenylacetate. Symbols: +, positive; –, negative.

Characteristic	1	2	3	4
Isolation source	Wetland sediment	Rivulet water	Wetland sediment	River sediment
Colony colour	Light orange	Light orange	Light yellow	Pale pink
Growth range (optimum) of				
Temperature (°C)	15–35 (30)	4–30 (25–30)	10–35 (30)	10–42 (30–37)
pH	6–7 (6)	6–7 (6)	6–7 (6)	5.5–8.5 (7)
NaCl (%)	0–0.5 (0)	0 (0)	0–0.5 (0)	0 (0)
Oxygen requirement	Strictly aerobic	Strictly aerobic	Facultative aerobic	Strictly aerobic
Nitrate reduction*	+	+	–	–
Hydrolysis* of				
Casein, starch	–	–	+	+
Gelatin	–	+	–	–
Tween 80	+	+	–	–
Enzyme activity* of				
Arginine dihydrolase	–	+	–	–
Assimilation* of				
d-Mannitol	–	+	–	+
d-Glucose, d-mannose	+	+	–	–
l-Arabinose, potassium gluconate, adipic acid, malic acid	–	–	–	+
*N*-Acetylglucosamine, d-maltose	–	+	–	–

* These data were obtained from this study under the same conditions.

## Chemotaxonomic characteristics

Respiratory isoprenoid quinones were extracted from strains AW1-3^T^ and AW1-7^T^ after cultivation in R2A broth at 30 °C for 2 days and analysed using an HPLC system (LC-20A, Shimadzu, Japan) equipped with a reversed-phase column (250×4.6 mm, Kromasil, Akzo Nobel, Netherlands) and a diode array detector (SPD-M20A, Shimadzu), following the method described by Minnikin *et al*. [36]. A methanol-isopropanol mixture (2:1, v/v) was used as the mobile phase at a flow rate of 1 ml min^−1^. For cellular fatty acid analysis, strains AW1-3^T^ and AW1-7^T^ and two reference strains were cultivated aerobically in R2A broth at their respective optimal temperatures and harvested during the exponential growth phase (OD_600_=0.7–0.8). Fatty acid methyl esters were prepared using the standard MIDI protocol (Sherlock Microbial Identification System, version 6.2B), including saponification, methylation and extraction steps. The resulting samples were analysed with a gas chromatograph (Hewlett Packard 6890, USA), and fatty acids were identified using the RTSBA6 database (Sherlock version 6.0B) [37]. For polar lipid analysis, cells of strains AW1-3^T^ and AW1-7^T^ were collected during the exponential phase, and polar lipids were extracted and separated by two-dimensional TLC as described by Minnikin *et al*. [38]. Specific spray reagents were used to detect lipid classes: 10% ethanolic molybdophosphoric acid for total lipids, ninhydrin for aminolipids, Dittmer–Lester reagent for phospholipids and *α*-naphthol/sulphuric acid for glycolipids. The presence of PE was confirmed using standard compounds (Sigma-Aldrich, USA).

MK-7 was identified as the sole respiratory isoprenoid quinone in strains AW1-3^T^ and AW1-7^T^, consistent with other members of the genus *Mucilaginibacter* [1,18]. The major cellular fatty acids (>10% of total fatty acids) in strain AW1-3^T^ were iso-C_15:0_ (37.4%) and summed feature 3 (comprising C_16: 1_* *ω*7c* and/or C_16:1_* *ω*6c*, 19.6%), while those in strain AW1-7^T^ were C_16:0_ (22.5%), summed feature 3 (17.8%), C_18:0_ (17.1%) and iso-C_15:0_ (12.9%) (Table S3). Although the overall fatty acid profiles of both strains were similar to those of closely related *Mucilaginibacter* type strains, notable differences were observed in the relative abundance of specific components. For instance, C_18: 0_ was present in high amounts in strain AW1-7^T^ but was found at very low levels in other strains. Both strains AW1-3^T^ and AW1-7^T^ contained PE as the major polar lipid (Fig. S3), consistent with other *Mucilaginibacter* species [1,18]. In addition, strain AW1-3^T^ contained two unidentified phospholipids, one unidentified aminophospholipid and two unidentified aminolipids, while strain AW1-7^T^ harboured one unidentified aminophospholipid and one unidentified polar lipid.

## Taxonomic conclusion

In conclusion, the phylogenetic, genomic, phenotypic, biochemical and chemotaxonomic data collectively support that strains AW1-3^T^ and AW1-7^T^ represent two distinct novel species within the genus *Mucilaginibacter*. Accordingly, the names *Mucilaginibacter aureus* sp. nov. and *Mucilaginibacter sediminis* sp. nov. are proposed for strains AW1-3^T^ and AW1-7^T^, respectively.

## Description of *Mucilaginibacter aureus* sp. nov.

*Mucilaginibacter aureus* (au’re.us. L. masc. adj. *aureus*, golden, referring to the orange colour of the strain).

Colonies on R2A agar are circular, convex and light orange in colour. Cells are Gram-stain-negative, strictly aerobic and non-motile rods lacking flagella, with no evidence of gliding motility. Growth occurs at 15–35 °C (optimum, 30 °C), pH 6.0–7.0 (optimum, pH 6.0) and in the presence of 0–0.5% (w/v) NaCl (optimum, 0%). Cells are oxidase- and catalase-positive. Nitrate is reduced to nitrite, but nitrogen gas is not produced. Indole production and glucose fermentation are negative. Tween 80 and aesculin are hydrolysed, whereas Tween 20, gelatin, casein, starch and tyrosine are not. *β*-Glucosidase and *β*-galactosidase activities are present, while arginine dihydrolase and urease activities are absent. d-Glucose and d-mannose are assimilated, but l-arabinose, d-mannitol, *N*-acetylglucosamine, d-maltose, potassium gluconate, caprate, adipate, malate, citrate and phenylacetate are not. MK-7 is the sole respiratory quinone. The major fatty acids (>10%) are iso-C_15:0_ and summed feature 3 (comprising C_16:1_* *ω*7c* and/or C_16:1_* *ω*6c*). The major polar lipids include PE, two unidentified phospholipids, one unidentified aminophospholipid and two unidentified aminolipids.

The type strain is AW1-3^T^ (=KACC 23848^T^=JCM 37500^T^), isolated from soil sediment of a reed wetland in the Republic of Korea. The genome size of the strain is 5509 kb, with a DNA G+C content of 43.9 mol%, as determined from its whole-genome sequence. The GenBank accession numbers for the 16S rRNA gene and whole-genome sequences are PQ270112 and CP170601, respectively.

## Description of *Mucilaginibacter sediminis* sp. nov.

*Mucilaginibacter sediminis* (se.di’mi.nis. L. gen. n. *sediminis*, of sediment).

Colonies on R2A agar are circular, convex and light yellow in colour. Cells are Gram-stain-negative, facultatively aerobic and non-motile rods lacking flagella, with no evidence of gliding motility. Growth occurs at 10–35 °C (optimum, 30 °C), pH 6.0–7.0 (optimum, pH 6.0) and in the presence of 0–0.5% (w/v) NaCl (optimum, 0%). Cells are oxidase- and catalase-positive. Nitrate is not reduced to nitrite. Indole production and glucose fermentation are negative. Casein, aesculin and starch are hydrolysed, whereas gelatin, Tween 20, Tween 80 and tyrosine are not. *β*-Glucosidase and *β*-galactosidase activities are present, while arginine dihydrolase and urease activities are absent. None of the following carbon sources is assimilated: d-glucose, l-arabinose, d-mannose, d-mannitol, *N*-acetylglucosamine, d-maltose, potassium gluconate, caprate, adipate, malate, citrate and phenylacetate. MK-7 is the sole respiratory quinone. The major fatty acids (>10% of the total) are C_16:0_, summed feature 3 (comprising C_16 :1_* ω*7*c* and/or C_16:1_* ω*6*c*), C_18:0_ and iso-C_15:0_. The major polar lipids are PE, one unidentified aminophospholipid, and one unidentified polar lipid.

The type strain is AW1-7^T^ (=KACC 23849^T^=JCM 37501^T^), isolated from soil sediment of a reed wetland in the Republic of Korea. The genome size of the strain is 7,410 kb, with a DNA G+C content of 43.2 mol%, as determined from its whole-genome sequence. The GenBank accession numbers for the 16S rRNA gene and whole-genome sequences are PQ270113 and CP170602, respectively.

## Supplementary material

10.1099/ijsem.0.007042 Uncited Fig. S1.

## Abbreviations

ANI: average nucleotide identity
CAZy: carbohydrate-active enzyme
dDDH: digital DNA–DNA hybridization
GTDB-Tk: Genome Taxonomy Database Toolkit
IMNGS: Integrated Microbial Next-Generation Sequencing
LB: Luria–Bertani
MA: marine agar
MK-7: menaquinone-7
ML: maximum likelihood
MP: maximum parsimony
NA: nutrient agar
NJ: neighbour-joining
PE: phosphatidylethanolamine
R2A: Reasoner’s 2A
TSA: tryptic soy agar
