# Supplementary material for: Mucilaginibacter aureus sp. nov. and Mucilaginibacter sediminis sp. nov., isolated from wetland soil
Source: Int J Syst Evol Microbiol. 2026 Jan 22;76(1):007042. doi: 10.1099/ijsem.0.007042 (PMC12828077; doi:10.1099/ijsem.0.007042)
Supplement: Uncited Fig. S1. [file ijsem-76-07042-s001.pdf]

Supplementary Information

Fig. S1. Maximum-likelihood (a) and maximum-parsimony (b) trees showing the phylogenetic relationships of strains AW1-3^{T} and AW1-7^{T} with closely related taxa based on 16S rRNA gene sequences. Bootstrap values (>70%) from 1000 replicates are indicated at the branch nodes. *Sphingobacterium spiritivorum* ATCC 33861^{T} (M58778) was used as the outgroup. Scale bars in panels A and B indicate the number of substitutions per nucleotide and total nucleotide substitutions, respectively.

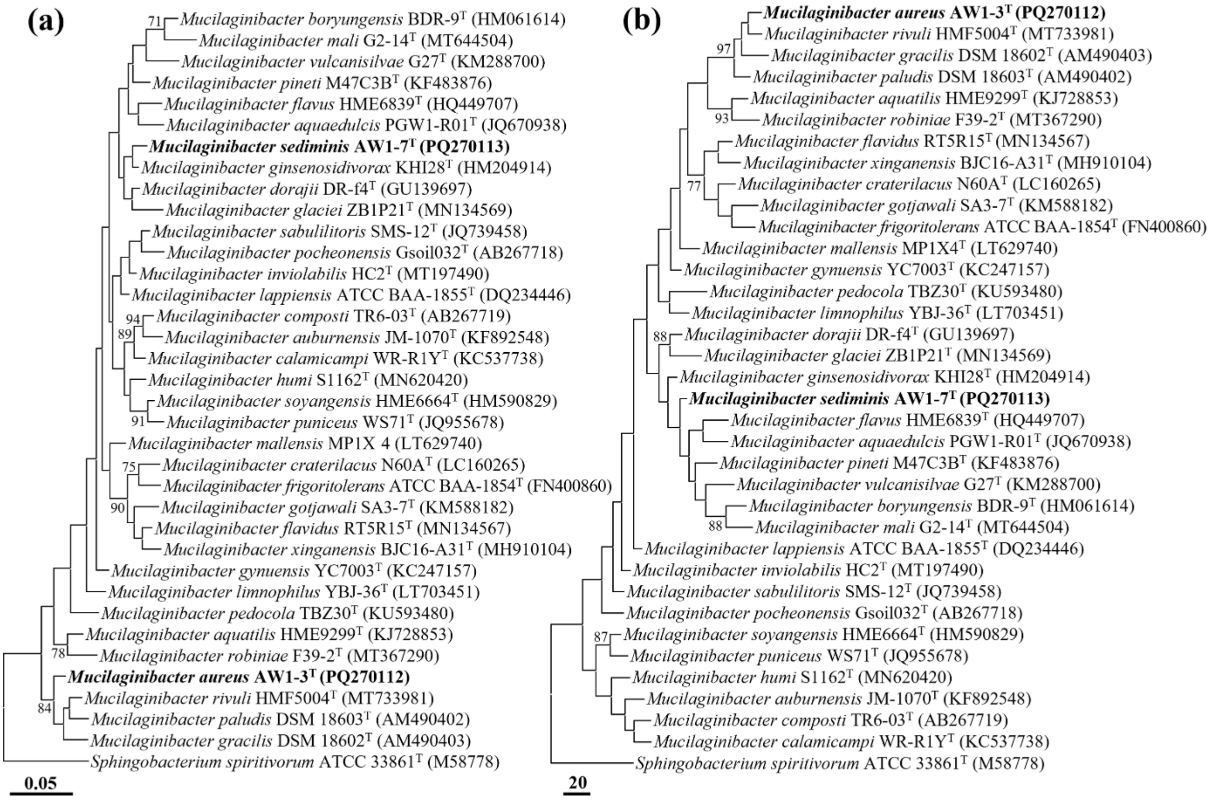

Fig. S2. Transmission electron micrographs showing the cellular morphology of strains AW1-3^{T} (a) and AW1-7^{T} (b), negatively stained with 2% uranyl acetate after cultivation on R2A agar at 30°C for 2 days. Scale bars: 1 μm.

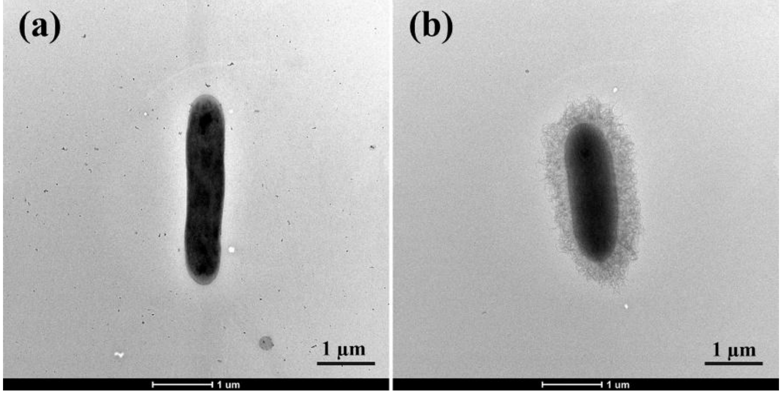

Fig. S3. Two-dimensional thin-layer chromatograms (TLC) showing the polar lipid of strains AW1-3^T and AW1-7^T. Solvent systems: (I) chloroform-methanol-water (65:25:4, v/v/v) and (II) chloroform-acetic acid-methanol-water (80:15:12:4, v/v/v/v). The TLC plates were sprayed with 10% ethanolic molybdophosphoric acid (a), ninhydrin (b), Dittmer-Lester (c), and α-naphthol/sulfuric acid (d) reagents for the detection of total polar lipids, aminolipids, phospholipids, and glycolipids, respectively. PE, phosphatidylethanolamine; PL, unidentified phospholipid; AL, unidentified aminolipid; APL, unidentified aminophospholipid; L, unidentified polar lipid.

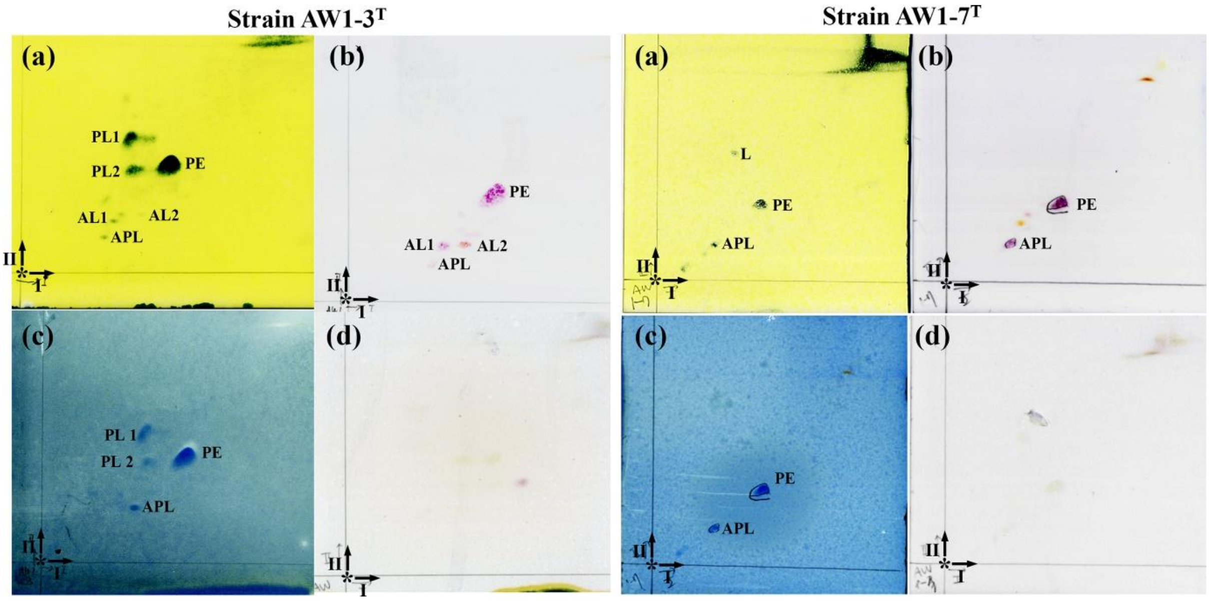

Table S1. Potential ecological distributions of strains AW1-3^T and AW1-7^T assessed by comparing their 16S rRNA gene sequences against metagenomic 16S rRNA amplicon datasets using the Integrated Microbial Next-Generation Sequencing (IMNGS) platform at a 99.0% sequence similarity threshold. "Matched no. (prevalence)" represents the number (percentage) of datasets containing sequences matching the 16S rRNA genes of strains AW1-3^T and AW1-7^T, and "ARA" denotes the average relative abundance of these sequences within the datasets. Prevalence values below 1.0% for both strains are not shown. –, not detected.

Habitats	No. of datasets	Strain AW1-3^T		Strain AW1-7^T	
		Matched no. (prevalence, %)	ARA (%)	Matched no. (prevalence, %)	ARA (%)
Ginkgo	275	173 (62.9)	2.904	53 (19.3)	0.10
Ascomycota	24	8 (33.3)	0.002	–	–
Onthophagus similis	5	1 (20.0)	0.0002	–	–
Crioceris duodecimpunctata	10	2 (20.0)	0.02	1 (10.0)	0.002
Cancer borealis	31	3 (9.7)	0.0003	9 (29.0)	0.009
Leaf litter	518	39 (7.5)	0.02	2 (0.4)	0.0003
Zea mays	45	3 (6.7)	0.01	–	–
Hordeum vulgare	30	2 (6.7)	0.002	16 (53.3)	0.29
Rhipicephalus sanguineus	39	2 (5.1)	0.0003	–	–
Glechoma hederacea	88	1 (1.1)	0.00004	10 (11.4)	0.0012
Bacteriastrum furcatum	25	1 (4.0)	0.00006	–	–
Glacier	247	8 (3.2)	0.002	10 (4.0)	0.003
Calliphora	36	1 (2.8)	0.001	–	–
Calliphora terraenovae	31	–	–	1 (3.2)	0.0001
Boechera stricta	1225	32 (2.6)	0.0006	19 (1.6)	0.0003
Fungus-growing ant	178	3 (1.7)	0.002	1 (0.6)	0.0001
Glycine max	61	1 (1.6)	0.0005	–	–
Dust	1018	14 (1.4)	0.0004	11 (1.1)	0.0004
Gorilla gorilla	73	1 (1.4)	0.0005	–	–
Solanum tuberosum	85	1 (1.2)	0.0003	–	–
Beta vulgaris	260	3 (1.2)	0.0004	3 (1.2)	0.00007
Soil crust	264	3 (1.1)	0.0003	–	–
Soil	67790	741 (1.1)	0.0007	357 (0.5)	0.0006
Urban	35	–	–	1 (2.9)	0.0006
Nematode	149	1 (0.7)	0.00002	4 (2.7)	0.002
Aedes albopictus	41	–	–	1 (2.4)	0.00002
Bos taurus	687	–	–	12 (1.7)	0.005
Reticulitermes flavipes	58	–	–	1 (1.7)	0.00009
Rhizosphere	14155	132 (0.9)	0.0007	165 (1.2)	0.002
Plant	12101	107 (0.9)	0.003	136 (1.1)	0.001

Table S2. Genome relatedness between strains AW1-3^T and AW1-7^T and their closely related type strains of the genus *Mucilaginibacter*

Taxa: 1, strain AW1-3^T (CP170601); 2, strain AW1-7^T (CP170602); 3, *M. rivuli* HMF5004^T (JAHXPQ000000000); 4, *M. ginsenosidivorax* KHI28^T (CP042437).

		dDDH† value (%)			
		**1**	**2**	**3**	**4**
ANI† value (%)	**1**	–	20.0	19.8	20.4
	**2**	70.9	–	20.1	36.6
	**3**	73.7	70.9	–	19.6
	**4**	70.7	88.6	70.9	–

†ANI, average nucleotide identity; dDDH, digital DNA-DNA hybridization.

Table S3. Comparison of cellular fatty acid compositions (%) of strains AW1-3^T and AW1-7^T and their closely related type strains of the genus *Mucilaginibacter*

Taxa: 1, strain AW1-3^T; 2, *M. rivuli* KCTC 82633^T; 3, strain AW1-7^T; 4, *M. ginsenosidivorax* KACC 14955^T. All data were obtained from this study. Data are expressed as percentages of the total fatty acids, and fatty acids constituting less than 1.0% in all strains are not shown. Major components (> 10.0%) are highlighted in bold. Symbols: tr, trace amount (< 1.0%); –, not detected.

Fatty acid	1	2	3	4
Saturated:				
$C_{12:0}$	tr	tr	1.8	tr
$C_{14:0}$	1.2	1.8	2.5	1.7
$C_{16:0}$	2.2	4.6	**22.5**	**12.5**
$C_{17:0}$	tr	–	1.0	–
$C_{18:0}$	tr	2.4	**17.1**	tr
Unsaturated:				
$C_{15:1}$ $\omega 5c$	1.0	1.1	tr	2.2
$C_{15:1}$ $\omega 6c$	tr	1.2	–	tr
$C_{16:1}$ $\omega 5c$	5.8	4.6	1.3	2.8
$C_{17:1}$ $\omega 8c$	1.9	tr	–	–
$C_{18:1}$ $\omega 9c$	–	tr	1.17	–
Branched:				
iso-$C_{15:0}$	**37.4**	**42.4**	**12.9**	**22.1**
anteiso-$C_{15:0}$	5.8	9.4	1.2	tr
iso-$C_{16:0}$	1.5	tr	–	–
Hydroxy:				
iso-$C_{12:0}$ 3-OH	tr	–	1.7	tr
iso-$C_{15:0}$ 3-OH	2.9	2.3	1.7	4.3
$C_{15:0}$ 2-OH	tr	tr	1.4	tr
$C_{16:0}$ 3-OH	tr	tr	1.3	–
iso-$C_{17:0}$ 3-OH	9.3	7.5	4.3	1.8
Summed feature*:				
3 ($C_{16:1}$ $\omega 7c$ and/or $C_{16:1}$ $\omega 6c$)	**19.6**	**14.4**	**17.8**	**44.9**
6 ($C_{19:1}$ $\omega 9c$ and/or $C_{19:1}$ $\omega 11c$)	tr	–	1.6	–
8 ($C_{18:1}$ $\omega 6c$ and/or $C_{18:1}$ $\omega 7c$)	–	–	1.7	–
9 (iso-$C_{17:1}$ $\omega 9c$ and/or $C_{16:0}$ 10-methyl)	2.5	1.2	2.1	tr

*Summed features are fatty acids that cannot be resolved reliably from another fatty acid using the chromatographic conditions chosen. The MIDI system groups these fatty acids together as one feature with a single percentage of the total.
